# CircSEMA6A upregulates PRRG4 by targeting MiR-520h and recruiting ELAVL1 to affect cell invasion and migration in papillary thyroid carcinoma

**DOI:** 10.20945/2359-4292-2021-0541

**Published:** 2024-02-23

**Authors:** Yachao Liu, Yunchao Xin, Xiaoling Shang, Zedong Tian, Gang Xue

**Affiliations:** 1 the First Affiliated Hospital of Hebei North University Department of Otolaryngol Head & Neck Surg Zhangjiakou City Hebei Peoples R China Department of Otolaryngol Head & Neck Surg, the First Affiliated Hospital of Hebei North University, Zhangjiakou City, Hebei, Peoples R China

**Keywords:** Papillary thyroid carcinoma, circSEMA6A, miR-520h, ELAVL1, PRRG4

## Abstract

**Objective:**

As the most prevalent type of thyroid malignancy, papillary thyroid carcinoma (PTC) accounts for over 80% of all thyroid cancers. Circular RNAs (circRNAs) have been found to regulate multiple cancers, including PTC.

**Materials and methods:**

Quantitative real-time polymerase chain reaction (qRT-PCR) and western blotting were used to analyse RNA and protein levels. Fluorescence in situ hybridization (FISH) was used to detect the distribution of the target genes. Functional experiments and animal experiments were implemented to analyse the biological functions of target genes *in vitro* and *in vivo*. Luciferase reporter, RNA pulldown, RNA binding protein immunoprecipitation (RIP) and mRNA stability assays were used to probe the underlying mechanisms.

**Results:**

CircSEMA6Awas found to be upregulated in PTC tissues and cells, and its circular structure was verified. CircSEMA6A promotes PTC cell migration and invasion. Moreover, circSEMA6A functions as a competing endogenous RNA (ceRNA) to upregulate proline rich and Gla domain 4 (PRRG4) expression by sponging microRNA-520h (miR-520h). CircSEMA6A recruits ELAV1 to stabilize PRRG4 mRNA and drives PTC progression via PRRG4.

**Conclusion:**

CircSEMA6A upregulates PRRG4 by targeting miR-520h and recruiting ELAVL1 to affect the invasion and migration of PTC cells, offering insight into the molecular mechanisms of PTC.

## INTRODUCTION

Over the last four decades, the incidence of thyroid cancer (TC) has increased across the globe (1). Papillary thyroid carcinoma (PTC), as the most frequent histotype, accounts for approximately 80%-90% of all TCs (2). Surgical resection, radioiodine therapy, and levothyroxine treatment have been used to treat PTC patients (3). PTC features an overall good prognosis, and its overall 5-year survival rate is as high as 97%. However, PTC can become life-threatening once it metastasizes or becomes invasive (4). Thus, clarifying the molecular mechanisms underlying the migration and invasion of PTC is of great significance to the improvement of PTC treatment.

CircRNA is a type of RNA with a covalently closed loop structure. It features a stable structure with tissue specificity. Many circRNAs play important roles in the occurrence and development of multiple human cancers (5,6). A large body of evidence has shown that many circRNAs participate in the regulation of PTC. For instance, circ_0005273 drives the progression of PTC by modulating SOX2 expression (7); circFNDC3B regulates PTC progression by targeting the miR-1178/TLR4 pathway (8); circ-PSD3 targets the miR-7-5p/METTL7B axis to aggravate PTC cell proliferation and invasion (9); and circBACH2 serves as a regulator in PTC by sequestering miR-139-5p to regulate LMO4 expression levels (10).

The role of circSEMA6A, the focus of this study, has rarely been reported. Its host gene, SEMA6A, can modulate multiple cancers according to previous studies. For instance, SEMA6A can act as a prognostic biomarker for glioblastoma (11). In addition, SEMA6A modulates the growth and survival of human melanoma cells (12). However, the roles of circSEMA6A and SEMA6Ahave never been reported in PTC.

PRRG4 was also a research object in our study. It has been found to facilitate the metastasis of breast cancer by recruiting NEDD4 and downregulating Robo1 (13). Moreover, Liu and cols. noted that PRRG4 can act as a potential prognostic marker in cholangiocarcinoma (14). However, the role of PRRG4 has never been explored in PTC.

In this study, we decided to investigate the roles of circSEMA6A and PRRG4 in PTC and their molecular mechanisms in cell migration and invasion. It is urgent to deepen our understanding of the mechanisms underlying PTC to develop treatments for PTC patients.

## MATERIALS AND METHODS

### Cell line culture and vector construction

Nthy-ori 3-1, IHH-4, TPC-1 and GLAG-66 cells were purchased from WHELAB (China). These cell lines were placed in RPMI-1640 medium supplemented with 10% FBS. As verified by Cellosaurus (https://web.expasy.org/cellosaurus/), all the cell lines were free of contamination. Full-length sequences of PRRG4 were inserted into the pcDNA3.1 vector for the construction of overexpression vectors, with the vector itself as a negative control (NC). Small interfering RNAs (siRNAs) against circSEMA6A and ELAVL1 as well as si-NC were constructed for interference.

### qRT-PCR

All RNAs were extracted from Nthy-ori 3-1, IHH-4, TPC-1 and GLAG-66 cells using TRIzol reagent. Afterwards, the total RNA was converted into cDNA by reverse transcription. All samples were assessed through qPCR using a qRT-PCR kit. The reaction conditions were as follows: 95°C for 10 min, 95 °C for 30s, and 60 °C for 30s. The results were quantified according to the 2^-ΔΔCt^ method. There were three replicates per condition.

### Western blotting

Total protein was isolated from PTC cells using a total protein extraction kit. After that, the samples were subjected to separation by SDS/PAGE and then transferred to polyvinylidene fluoride (PVDF) membranes. Afterwards, these membranes were blocked with 5% skim milk for 2 h at room temperature. Subsequently, they were subjected to overnight incubation with the primary antibodies at 4 °C, followed by 1 h of incubation with the secondary antibodies at room temperature. Finally, images we retaken and assessed. We used β-actin as our internal reference. The primary antibody used included anti-ELAVL1. There were three replicates per condition.

### Transwell assay

Transwell chambers coated with or without Matrigel were used to test the invasion and migration of transfected PTC cells. The transfected cells were plated into the upper chamber with the addition of 0.2 mL serum-free medium, while the lower chamber was supplemented with 0.6 mL medium containing 10% FBS. After 24 h, the cells in the upper chamber were removed. The PTC cells left in the lower chambers were counted using a microscope with the help of 0.2% crystal violet solution for staining. There were three replicates per condition.

### Wound healing assay

The wound healing assay was carried out to detect PTC cell migration. A pipette was used to scratch the cell layers. The average width of the wound was quantified 24 h later. There were three replicates per condition.

### FISH

PTC cells were subjected to fixation in 4% PFA for 15 min, and then permeabilization of the cells was conducted using 0.5% Triton X-100 for 15 min at 4 °C. Next, the digoxigenin (DIG)-labelledcircSEMA6A probe or control probe was incubated with PTC cells for 4 h at 55 °C. The signal was detected via Hoechst-conjugated anti-digoxin antibodies. A laser confocal microscope was employed to capture images. Cell nuclei were counterstained with DAPI. There were three replicates per condition.

### RIP assay

Magnetic beads were used for conjugation with anti-IgG, anti-AGO2 or anti-ELAVL1. PTC cells were lysed for the assay. The cell lysate was incubated with the conjugated magnetic beads. The precipitated RNAs were subjected to extraction and assessed by qPCR. There were three replicates per condition.

### RNA pulldown assay

The structure buffer was added to 1 μg of biotin-labelled circSMEA6A/miR-520h. Then, biotinylated RNAs were heated at 95 °C for 2 min, ice-bathed for 3 min, and left for 30 min at room temperature. Afterwards, 15 μL streptavidin beads were incubated with biotin-labelled and denatured RNA for 2h at 4 °C. Three groups (input, Bio-NC, Bio-circSMEA6A/miR-520h) were set up, and PTC cells in each group were lysed. The magnetic beads were mixed with the cell lysate, followed by overnight incubation at 4 °C. After incubation, RNA was extracted, followed by qRT-PCR and western blot analyses. There were three replicates per condition.

### Luciferase reporter assay

To perform the assay, pmirGLO-circSMEA6A/PRRG4 3’UTR-Wt was constructed by subcloning the sequences of circSMEA6A/PRRG4 3’UTR into the pmirGLO reporter vector. Similarly, the circSMEA6A/PRRG4 3’UTR containing mutated binding sites in the miR-520h seed region was inserted into the pmirGLO vector to create pmirGLO-circSMEA6A/PRRG4 3’UTR-Mut. PTC cells were placed into 96-well plates. These reporter vectors were cotransfected with miR-520hmimics or NC mimics into PTC cells. The vectors themselves were used as the negative controls. The luciferase activity was tested with Renilla luciferase as the internal reference. There were three replicates per condition.

### Animal experiments

BALB/c nude mice (4 weeks old; female) were divided into three groups, with 3 mice in each group. These animal experiments were permitted by the Ethics Committee of the First Affiliated Hospital of Hebei North University. GLAG-66 cells (1 × 10^6^) stably transfected with si-NC, si-circSEMA6A-1 or si-circSEMA6A-2 were subcutaneously injected into the right flanks of mice. From day 7 to day 28, the tumour size was measured every 3 days. On day 28, the mice were sacrificed. The tumours were removed and weighed.

### mRNA stability assay

For this assay, 50 mMα-amanitin was used to treat PTC cells to inhibit RNA transcription. qRT-PCR was used to examinePRRG4and β-actin expression levels at 0, 6, 12, 18 and 24 h. 18S rRNA, which is not affected by α-amanitin, served as the internal reference. There were three replicates per condition.

### Statistical analysis

Student’s t test and one-way/two-way analysis of variance (ANOVA) were applied to assess differences between groups. A P value lower than 0.05 was indicative of statistical significance.

## RESULTS

### CircSEMA6A, featuring a circular structure, is upregulated in PTC

**Figure 1:**
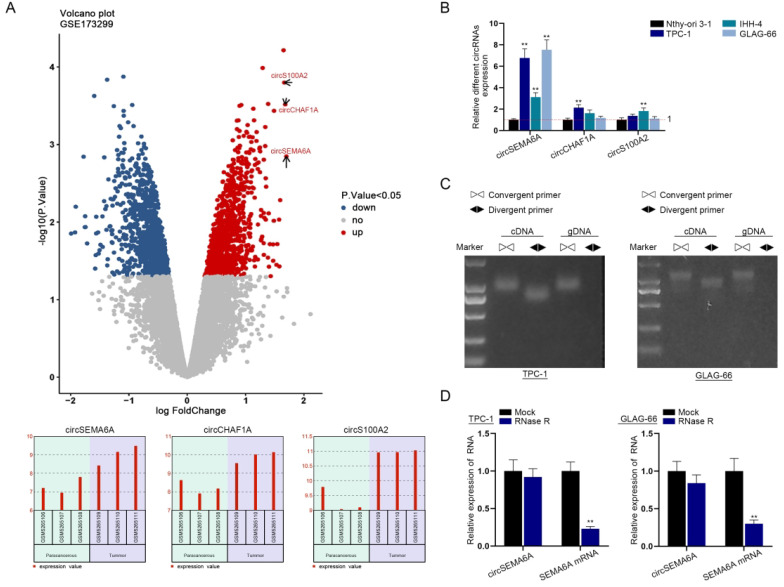
CircSEMA6A, featuring a circular structure, is upregulated in PTC.

We retrieved the Gene Expression Omnibus (GEO) dataset GSE173299 to screen circRNAs that were differentially overexpressed in PTC tissues relative to adjacent tissues. As indicated in the volcano plot, we identified three circRNAs, circRNA_103923, circRNA_405716 and circRNA_014234 (Figure 1A). The host genes of circRNA_103923, circRNA_405716 andcircRNA_014234 are SEMA6A, CHAF1A and S100A2, respectively. Hence, they are termed circSEMA6A, circCHAF1A and circS100A2. According to a literature review, circCHAF1A is overexpressed in glioma (15), while circSEMA6A and circS100A2 have never been reported to be involved in diseases. Next, we conducted qRT-PCR to examine the expression levels of three candidates in a human thyroid follicular epithelial cell line (Nthy-ori 3-1) and PTC cell lines (IHH-4, TPC-1 and GLAG-66). The results revealed that circSEMA6A was most overexpressed in PTC cell lines (Figure 1B). Therefore, circSEMA6A was selected as the focus of our study. Moreover, due to the relatively high expression of circSEMA6A, TPC-1 and GLAG-66 cells were chosen for follow-up assays. Next, PCR-agarose gel electrophoresis was performed to verify the circular structure of circSEMA6A. As indicated in Figure 1C, circSEMA6A was amplified by convergent primers and divergent primers in complementary DNA (cDNA), but in genomic DNA (gDNA), it was only amplified by convergent primers. This verified the circular structure of circSEMA6A. For further verification, qRT-PCR was implemented to determine circSEMA6A and SEMA6A mRNA levels after treatment with ribonuclease R (RNase R). SEMA6A mRNA was digested by RNase R, while circSEMA6A expression remained almost unchanged (Figure 1D). The results further validated the circular structure of circSEMA6A, as the structure of circRNA is more stable than that of linear RNA. Taken together, these findings indicate that circSEMA6A, featuring a circular structure, is upregulated in PTC.

**A**) The GSE173299 dataset was used to screen out the circRNAs most upregulated in PTC tissues. (**B**) The expression levels of circSEMA6A, circCHAF1A and circS100A2 were investigated by qRT-PCR in Nthy-ori 3-1, IHH-4, TPC-1 and GLAG-66 cells. (**C**) PCR-agarose gel electrophoresis in PTC cells verified the circular structure of circSEMA6A. (**D**) The expression levels of circSEMA6A and SEMA6A mRNA were evaluated by qRT-PCR in PTC cells after RNase R treatment. Student’s t test and one-way ANOVA were applied for statistical analysis. **P < 0.01.

### CircSEMA6A facilitates PTC progression in vitro and in vivo

**Figure 2:**
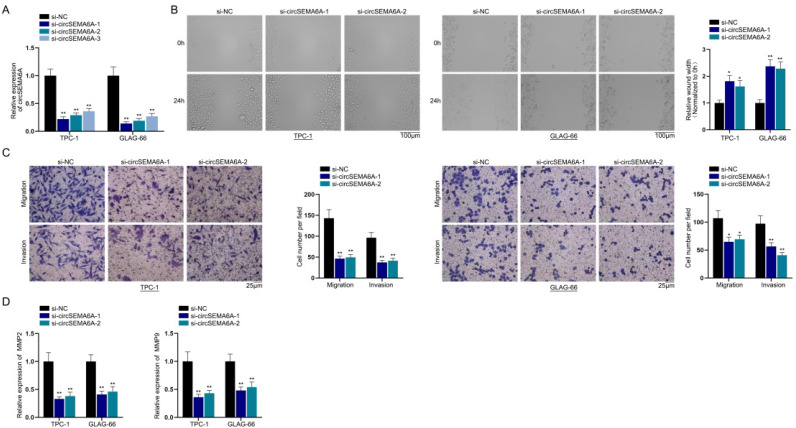
CircSEMA6A facilitates PTC cell migration and invasion.

Next, we performed functional assays to investigate the influence of circSEMA6A on PTC cell migration and invasion. First, we performed qRT-PCR to evaluate the expression of circSEMA6Aafter si-circSEMA6A-1/2/3 transfection. These plasmids suppressed the expression of circSEMA6A (Figure 2A). Owing to their higher efficiency, si-circSEMA6A-1 and si-circSEMA6A-2 were chosen for the following experiments. Afterwards, we performed a wound healing assay in PTC cells and found that the wound width was increased after the transfection of si-circSEMA6A-1/2 (Figure 2B). Transwell assays were implemented in PTC cells to detect the impacts of circSEMA6A on cell migration and invasion. The number of migrated cells and invaded cells was reduced after circSEMA6A depletion (Figure 2C). In addition, we performed qRT-PCR to evaluate the mRNA levels of the invasion-related proteins MMP2 and MMP9 after knockdown of circSEMA6A. From the results, we found that MMP2 and MMP9 were downregulated by circSEMA6Aknockdown (Figure 2D). Additionally, we conducted animal experiments to determine the functions of circSEMA6A *in vivo*. As displayed by the growth curve, tumour growth was suppressed after circSEMA6A knockdown. After the resection of tumours from mice, we measured the volume and weight of tumours. CircSEMA6A interference led to a decrease in tumour volume and weight. In summary, circSEMA6A aggravates PTC cell progression *in vitro* and *in vivo*.

(**A**) The efficiency of si-circSEMA6A-1/2/3 was determined by qRT-PCR in PTC cells. (**B-C)** Wound healing and transwell assays were implemented in PTC cells to detect cell migration and invasion after the depletion of circSEMA6A. (**D**) MMP2 and MMP9 mRNA levels were examined by qRT-PCR in PTC cells after the depletion of circSEMA6A. One-way ANOVA was used for statistical analysis. *P < 0.05, **P < 0.01.

### CircSEMA6A sponges miR-520h via ceRNA mode

**Figure 3:**
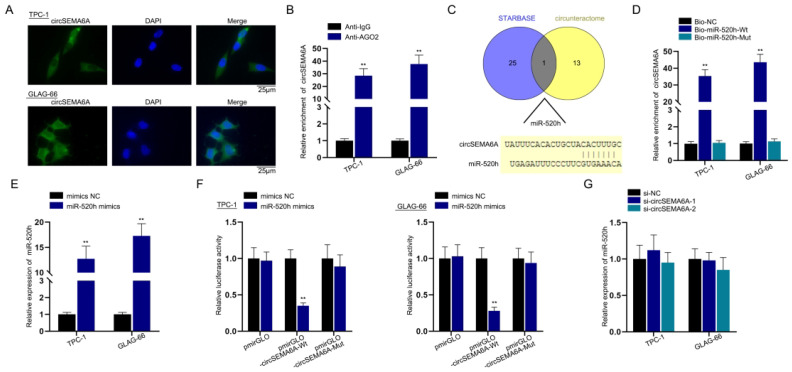
CircSEMA6A sponges miR-520h via the ceRNA mechanism.

We next probed the regulatory mechanisms of circSEMA6A. We first conducted a FISH assay to detect the subcellular distribution of circSEMA6A in PTC cells. As indicated in Figure 3A, circSEMA6A was located in both the cytoplasm and nucleus of PTC cells but mainly in the cytoplasm. Moreover, a RIP assay was performed to show the enrichment of circSEMA6A in AGO2 complexes (Figure 3B). This indicated its existence in the RNA-induced silencing complex (RISC), which is the major workplace for miRNAs. Based on the results of Figure 3A and Figure 3B, we hypothesized that circSEMA6A functions as a ceRNA. Subsequently, we utilized the starBase database (http://starbase.sysu.edu.cn/) and CircInteractome (https://circinteractome.nia.nih.gov/) to forecast miRNA binding with circSEMA6A. As indicated in the Venn diagram, miR-520h was predicted as a binding partner by both starBase and CircInteractome. Moreover, sequences of the binding site between miR-520h and circSEMA6A were obtained (Figure 3C). To prove the interaction between circSEMA6A and miR-520h, we carried out an RNA pulldown assay to determine the enrichment of circSEMA6A in samples pulled down by Bio-miR-520h-Wt and Bio-miR-520h-Mut (sequences shown in Figure 3C). The results revealed that circSEMA6A was most abundant in the Bio-miR-520h-Wt group, verifying the interaction (Figure 3D). We conducted qRT-PCR to probe the miR-520h expression level after miR-520h mimic transfection and found that miR-520h expression was upregulated (Figure 3E). Afterwards, a luciferase reporter assay was conducted to further validate the interaction between circSEMA6A and miR-520h. The results showed that the cotransfection of miR-520h mimics reduced pmirGLO-circSEMA6A-Wt luciferase activity, while that of pmirGLO-circSEMA6A-Mut (designed by the sequences shown in Figure 3C) remained unchanged (Figure 3F). We then implemented qRT-PCR to investigate the expression of miR-520h after the knockdown of circSEMA6A. The results revealed that miR-520h expression remained unchanged after circSEMA6Aknockdown (Figure 3G). Next, we carried out assays to examine the influence of miR-520h on PTC cell progression. Wound healing, Transwell and qRT-PCR assays revealed that PTC cell migration and invasion were hampered after miR-520h overexpression. Taken together, these findings indicate that circSEMA6A sponges miR-520h via the ceRNA mode.

(**A)** FISH detected the subcellular location of circSEMA6A in PTC cells. (**B**) RIP in PTC cells was performed to detect the enrichment of circSEMA6A in the anti-AGO2 group. (**C)** Venn diagram showing the bioinformatics predictions. The blue part indicates the miRNAs forecasted by the starBase database (http://starbase.sysu.edu.cn/), while the yellow part indicates the miRNAs forecasted by CircInteractome (https://circinteractome.nia.nih.gov/). The binding sites between circSEMA6A and miR-520hare shown. (**D**) RNA pulldown assay was implemented in PTC cells to validate the interaction between circSEMA6A and miR-520h. (**E**) The efficiency of miR-520h mimics was assessed by qRT-PCR in PTC cells. (**F**) A luciferase reporter assay was conducted in PTC cells to verify the interaction between circSEMA6A and miR-520h. (**G**) MiR-520h expression levels were examined by qRT-PCR in PTC cells after silencing circSEMA6A. Student’s t test and one-way/two-way ANOVA were utilized for statistics. **P < 0.01.

### CircSEMA6A sponges miR-520h to upregulate PRRG4 expression

**Figure 4:**
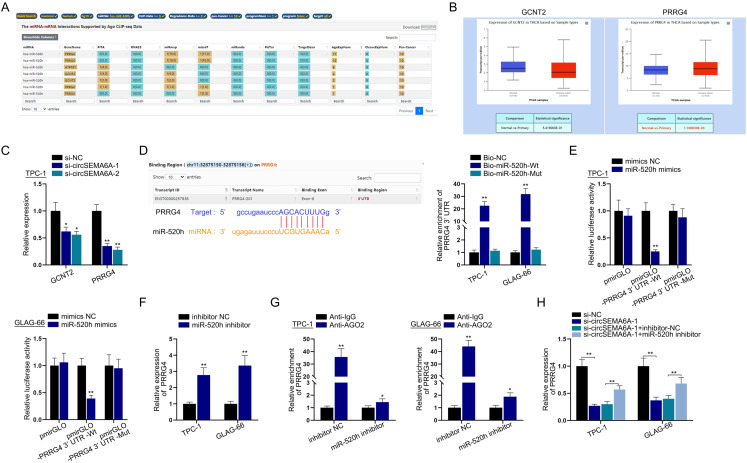
CircSEMA6A sponges miR-520h to upregulate PRRG4 expression.

We next probed the downstream mRNA targeted by miR-520h. We used starBase to predict the mRNA binding to miR-520h under certain conditions (CLIP-Data>=5, pan-Cancer>=10). PRRG4, ATP1B1 and GCNT2 were selected as candidates (Figure 4A). According to a literature review, PRRG4 is highly expressed in breast cancer and promotes its metastasis (13); ATP1B1 is expressed at low levels in PTC tissues (16), which is not in line with our research and thus was excluded; and GCNT2 can induce the epithelial-mesenchymal transition of oesophageal squamous cell carcinoma cells and enhance cell migration and invasion (17). There is no research report on PRRG4 and GCNT2 in PTC. Therefore, we first analysed the expression of PRRG4 and GCNT2 in PTC tissues by using bioinformatics. UALCAN predictions (http://ualcan.path.uab.edu/) showed that only PRRG4 was significantly overexpressed in PTC tissues (Figure 4B). Furthermore, we implemented qRT-PCR to examine PRRG4 and GCNT2 expression levels in TPC-1 cells after the knockdown of circSEMA6A and found that PRRG4 expression was much lower than GCNT2 expression (Figure 4C). Based on the results of Figure 4B-C, we selected PRRG4 as the focus of our study. We obtained the binding sites between PRRG4 and miR-520h from starBase and then performed an RNA pulldown assay in PTC cells to evaluate the binding between PRRG4 and miR-520h. The results showed that the PRRG4 3’UTR was more enriched in the Bio-miR-520h-Wt group than in the Bio-miR-520h-Mut group, verifying the interaction (Figure 4D). Afterwards, a luciferase reporter assay was conducted in PTC cells to further validate the interaction between PRRG4 and miR-520h. The results revealed that the cotransfection of miR-520h mimics reduced pmirGLO-PRRG4 3’UTR-Wt luciferase activity, while that of pmirGLO-PRRG4 3’UTR-Mut (designed by the sequences shown in Figure 4D) remained unchanged (Figure 4E). Next, we conducted qRT-PCR in PTC cells to examine the expression of PRRG4 after transfection with the miR-520h inhibitor. The results showed that PRRG4 was overexpressed with miR-520h inhibitor treatment, indicating the successful construction of the miR-520h inhibitor (Figure 4F). Subsequently, a RIP assay in PTC cells was used to detect the enrichment of PRRG4 in the anti-AGO2 group after the knockdown of miR-520h. PRRG4 enrichment was decreased after miR-520h knockdown (Figure 4G). Next, the regulatory axis of circSEMA6A, PRRG4 and miR-520h was explored by rescue experiments. We carried out qRT-PCR to evaluate PRRG4 expression in PTC cells after transfection with si-NC, si-circSEMA6A-1, si-circSEMA6A-1+inhibitor-NC or si-circSEMA6A-1+miR-520h inhibitor. The results showed that PRRG4 expression was decreased withcircSEMA6A knockdown and was then partially rescued by miR-520h interference (Figure 4H). Taken together, circSEMA6A sponges miR-520h to upregulate PRRG4 expression.

(**A**) The mRNAs interacting with miR-520h were predicted by starBase. (**B**) UALCAN (http://ualcan.path.uab.edu/) predicted the expression of PRRG4 and GCNT2 in PTC tissues. (**C**) The expression levels of PRRG4 and GCNT2 were probed by qRT-PCR in TPC-1 cells after the knockdown of circSEMA6A. (**D**) RNA pulldown assays were conducted to evaluate the binding between the PRRG4 3’UTR and miR-520h. The binding sites between PRRG4 and miR-520h were obtained from starBase. (**E**) A luciferase reporter assay was conducted in TPC-1 and GLAG-66 cells to assess the interaction between the PRRG4 3’UTR and miR-520h. (**F**) The efficiency of the miR-520h inhibitor was determined by qRT-PCR in TPC-1 and GLAG-66 cells. (**G**) RIP assays were conducted in PTC cells to detect the enrichment of PRRG4 in the anti-AGO2 group after the knockdown of miR-520h. (**H**) PRRG4 expression levels were determined by qRT-PCR in TPC-1 and GLAG-66 cells after transfection with si-NC, si-circSEMA6A-1, si-circSEMA6A-1+inhibitor-NC or si-circSEMA6A-1+miR-520h inhibitor. Student’s t test and one-way/two-way ANOVA were applied for statistical analysis. *P < 0.05; **P < 0.01.

### CircSEMA6A recruits ELAVL1 to stabilize PRRG4 mRNA

**Figure 5:**
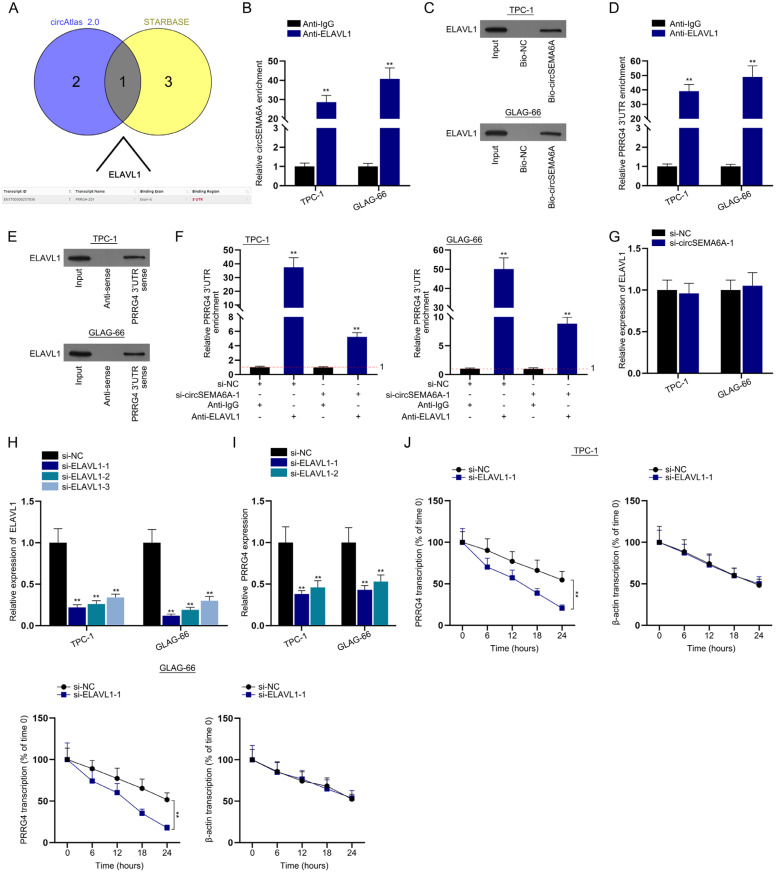
CircSEMA6A recruits ELAVL1 to stabilize PRRG4 mRNA.

As indicated in the rescue experiments, PRRG4 expression was only partially rescued by miR-520h interference. Therefore, we speculated that circSEMA6A can also regulate the expression of PRRG4 through other mechanisms. It has been reported that circRNAs regulate downstream targets by recruiting RNA binding proteins (RBPs) (18,19). Hence, we conducted experiments to study whether circSEMA6A regulates the expression of PRRG4 by recruiting RBPs. First, we performed bioinformatics analysis to identify the RBPs interacting with circSEMA6A. ELAVL1 was predicted by both starBase and circAtlas 2.0 (http://circatlas.biols.ac.cn/), as indicated in the Venn diagram (Figure 5A). ELAVL1 can stabilize mRNA by binding to its 3’UTR (20). We performed a RIP assay to investigate the interaction between ELAVL1 and circSEMA6A. The results revealed that circSEMA6A was abundant in the samples precipitated by anti-ELAVL1 (Figure 5B). For further validation, we implemented an RNA pulldown assay and found that ELAVL1 protein was enriched in the Bio-circSEMA6A group (Figure 5C). Afterwards, we performed RIP and RNA pulldown to probe the binding of the PRRG4 3’UTR to ELAVL1. The results revealed that the PRRG4 3’UTR was abundant in the samples precipitated by anti-ELAVL1 and that the ELAVL1 protein was enriched in PRRG4 3’UTR-sense (Figure 5D-E). We performed a RIP assay to detect the influence of circSEMA6A on the interaction between the PRRG4 3’UTR and ELAVL1. CircSEMA6A decreased the ELAVL1-induced enrichment of the PRRG4 3’UTR (Figure 5F). Next, qRT-PCR was implemented to examine ELAVL1 expression levels after interference with circSEMA6A. CircSEMA6A overexpression did not affect the expression of ELAVL1 (Figure 5G). We performed qRT-PCR to investigate the expression of ELAVL1 after si-ELAVL1-1/2/3 transfection. These plasmids suppressed the expression of ELAVL1 (Figure 5H). Owing to the higher efficiency, we chose si-ELAVL1-1 and si-ELAVL1-2 for the ensuing assays. Next, we conducted qRT-PCR to examine the expression of PRRG4 after the knockdown of ELAVL1. ELAVL1 knockdown led to a decrease inPRRG4 expression (Figure 5I). Finally, we conducted qRT-PCR to assess the stability of PRRG4 in PTC cells treated withα-amanitin. The results showed that the mRNA stability of PRRG4 was decreased after ELAVL1knockdown (Figure 5J). Taken together, these findings indicate that circSEMA6A recruits ELAVL1 to stabilize PRRG4 mRNA.

(**A**) Venn diagram showing the bioinformatics predictions. The yellow part indicates the miRNAs predicted by the starBase database, while the blue part indicates the miRNAs forecasted by circAtlas 2.0 (http://circatlas.biols.ac.cn/). (**B-C**) RIP and RNA pulldown assays were used to detect the interaction between ELAVL1 and circSEMA6A in PTC cells. (**D-E**) RIP and RNA pulldown assays in PTC cells were used to detect the interaction between ELAVL1 and the PRRG4 3’UTR in PTC cells. (**F**) RIP assay detecting the interaction between ELAVL1 and the PRRG4 3’UTR after circSEMA6A knockdown in TPC-1 and GLAG-66 cells. (**G**) ELAVL1 expression levels were determined by qRT-PCR in PTC cells after circSEMA6A knockdown. (**H**) The efficiency of si-ELAVL1-1/2/3 was determined by qRT-PCR in PTC cells. (**I**) PRRG4 expression levels were investigated by qRT-PCR in PTC cells after ELAVL1knockdown. (**J**) The stability of PRRG4 was assessed by qRT-PCR in PTC cells after ELAVL1knockdown. Student’s t test and one-way ANOVA were utilized for statistical analysis. **P < 0.01.

### CircSEMA6A regulates PTC cell migration and invasion via PRRG4

**Figure 6:**
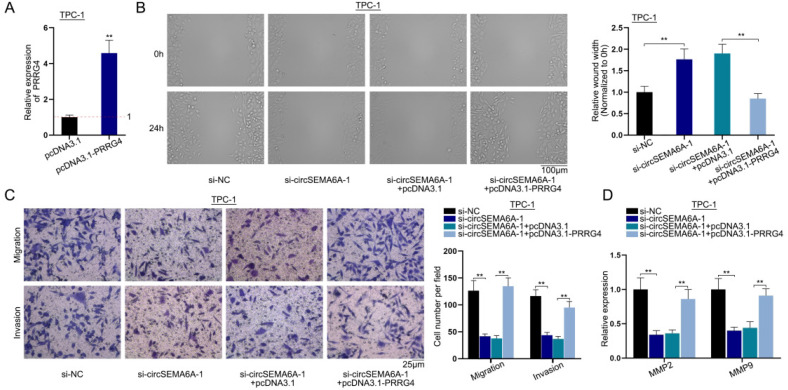
CircSEMA6A regulates PTC cell migration and invasion via PRRG4.

Our results clearly indicated that circSEMA6A upregulates PRRG4 by targeting miR-520h and recruiting ELAVL1, so we next designed rescue experiments for further validation. We carried out qRT-PCR to assess PRRG4 expression levelsafterpcDNA3.1-PRRG4 transfection. pcDNA3.1-PRRG4enhanced the expression of PRRG4 (Figure 6A). We next performed a wound healing assay in TPC-1 cells after the transfection of si-NC, si-circSEMA6A-1, si-circSEMA6A-1+pcDNA3.1 or si-circSEMA6A-1+pcDNA3.1-PRRG4 and found that the increase in wound width induced by circSEMA6A was counteracted after the transfection of pcDNA3.1-PRRG4 (Figure 6B). Transwell assays were implemented in TPC-1 cells to determine cell migration and invasion after transfection. The number of migrated cells and invaded cells was reduced by circSEMA6A knockdown, and this change was reversed by PRRG4 overexpression (Figure 6C). We then performed qRT-PCR to evaluate the mRNA levels of MMP2 and MMP9 after transfection. From the results, we found that MMP2 and MMP9 were downregulated by circSEMA6A knockdown, this effect was reversed byPRRG4 overexpression (Figure 6D). In summary, circSEMA6A regulates PTC cell migration and invasion via PRRG4.

(**A**) The efficiency of pcDNA3.1-PRRG4 was assessed by qRT-PCR in TPC-1 cells. (**B-C**) Wound healing and transwell assays were conducted in TPC-1 cells to detect cell migration and invasion after the transfection of si-NC, si-circSEMA6A-1, si-circSEMA6A-1+pcDNA3.1 or si-circSEMA6A-1+pcDNA3.1-PRRG4. (**D**) MMP2 and MMP9 mRNA levels were measured by qRT-PCR in TPC-1 cells after the transfection of si-NC, si-circSEMA6A-1, si-circSEMA6A-1+pcDNA3.1 or si-circSEMA6A-1+pcDNA3.1-PRRG4. Student’s t test and one-way ANOVA were used for statistical analysis. **P < 0.01.

## DISCUSSION

Previous studies indicated that circRNAs can modulate PTC cell migration and invasion. CircFAT1 (e2) is overexpressed in PTC cells and facilitates PTC cell migration and invasion (21). Circ_007293 is abundant in PTC cells and drives cell migration and invasion (22). In our study, we used the GEO database to screen out circRNAs overexpressed in PTC tissues. Afterwards, we carried out qRT-PCR to evaluate the expression of circRNAs in PTC cell lines and identified circSEMA6A, which was overexpressed in PTC cell lines. Furthermore, we used wound healing, Transwell and qRT-PCR assays to validate that circSEMA6A promotes PTC cell migration and invasion. Animal experiments verified the functions of circSEMA6A *in vivo*.

Previously, ceRNA networks have been widely explored in PTA. Circ-PRKCI has been reported to propel PTC cell progression via themiR-335/E2F3 ceRNA axis (23); circFOXM1 plays a regulatory role in PTC by sequestering miR-1179 to regulate the expression of HMGB1 (24); and circRPS28 regulates cell proliferation and migration by functioning as a ceRNA to target the miR-345-5p/FZD8 regulatory axis (25). In line with previous studies, circSEMA6A was proven to function as a ceRNA based on the results of FISH and RIP assays. We used bioinformatics to predict the downstream target miRNAs and mRNAs of circSEMA6A, miR-520h and PRRG4. MiR-520h can be sponged by PART1 to overexpress CTNNB1, thereby regulating the development of colorectal cancer (26). CircCSPP1 enhances prostate cancer cell progression by sequestering miR-520h to regulate EGR1 (27). MiR-520h overexpression hampers pancreatic cancer cell migration and invasion and is targeted by LINC00657 to modulate CKS1B in pancreatic cancer cells (28). PRRG4 is the downstream mRNA of miR-1299 in oesophageal squamous cell carcinoma (29). In this study, we carried out mechanistic assays, proving that circSEMA6A acts as a sponge for miR-520h and that miR-520h can bind to PRRG4. Functional experiments proved that miR-520h overexpression hinders PTC cell migration and invasion. Rescue experiments revealed that miR-520h knockdown partially rescued the inhibitory effects of circSEMA6A knockdown on PRRG4 expression and that circSEMA6A promoted PTC cell migration and invasion via PRRG4. In summary, circSEMA6A functions as a ceRNA to upregulatePRRG4 expression by sequestering miR-520h, thereby facilitating PTC cell migration and invasion.

The potential RBPs of circSEMA6A and ELAVL1 were predicted by bioinformatics. It has been reported that ELAVL1 interacts with TIMM44 mRNA to regulate its stability in ovarian cancer (30). In addition, ELAVL1 can interact with thePTBP1 3’UTR to enhance the stabilization of PTBP1incolorectal cancer (31). We investigated whether ELAVL1 can modulate the stability of PRRG4 mRNA in PTC cells. RIP and RNA pulldown assays were used to detect the interaction between ELAVL1 and circSEMA6A and ELAVL1 and the PRRG4 3’UTR. We then performed a RIP assay and proved that circSEMA6A inhibits the interaction between ELAVL1 and the PRRG4 3’UTR. As indicated by the mRNA stability assay, ELAVL1 knockdown led to a decrease in PRRG4 mRNA stability.

In conclusion, circSEMA6A upregulates PRRG4 by targeting miR-520h and recruiting ELAVL1 to promote PTC cell invasion and migration. The current study identified a novel circRNA whose host gene is SEMA6A, which promotes PTC cell progression. Furthermore, our study first investigated the mechanisms by whichcircSEMA6A exerts its functions. The findings in our study may aid in the development of PTC treatments. Investigating the underlying molecular mechanisms of circSEMA6Ain PTC might contribute to the treatment of PTC patients. However, clinicopathological analysis is needed to explore its clinical applicability. We will study the association of circSEMA6A with PTC patients in our future study.
